# TNFα: TNFR1 signaling inhibits maturation and maintains the pro-inflammatory programming of monocyte-derived macrophages in murine chronic granulomatous disease

**DOI:** 10.3389/fimmu.2024.1354836

**Published:** 2024-02-09

**Authors:** Sophie L. Gibbings, Kelsey C. Haist, Elizabeth F. Redente, Peter M. Henson, Donna L. Bratton

**Affiliations:** ^1^ Department of Pediatrics, National Jewish Health, Denver, CO, United States; ^2^ Department of Medicine, University of Colorado Denver, Aurora, CO, United States; ^3^ Department of Immunology and Microbiology, University of Colorado Denver, Aurora, CO, United States; ^4^ Department of Pediatrics, University of Colorado Denver, Aurora, CO, United States

**Keywords:** tumor necrosis factor, monocyte-derived macrophages, hyperinflammation, chronic granulomatous disease (CGD), TNF inhibitor, macrophage programming

## Abstract

**Introduction:**

Loss of NADPH oxidase activity results in proinflammatory macrophages that contribute to hyperinflammation in Chronic Granulomatous Disease (CGD). Previously, it was shown in a zymosan-induced peritonitis model that gp91^phox-/-^ (CGD) monocyte-derived macrophages (MoMacs) fail to phenotypically mature into pro-resolving MoMacs characteristic of wild type (WT) but retain the ability to do so when placed in the WT milieu. Accordingly, it was hypothesized that soluble factor(s) in the CGD milieu thwart appropriate programming.

**Methods:**

We sought to identify key constituents using *ex vivo* culture of peritoneal inflammatory leukocytes and their conditioned media. MoMac phenotyping was performed via flow cytometry, measurement of efferocytic capacity and multiplex analysis of secreted cytokines. Addition of exogenous TNFα, TNFα neutralizing antibody and TNFR1-/- MoMacs were used to study the role of TNFα: TNFR1 signaling in MoMac maturation.

**Results:**

More extensive phenotyping defined normal MoMac maturation and demonstrated failure of maturation of CGD MoMacs both *ex vivo* and *in vivo*. Protein components, and specifically TNFα, produced and released by CGD neutrophils and MoMacs into conditioned media was identified as critical to preventing maturation. Exogenous addition of TNFα inhibited WT MoMac maturation, and its neutralization allowed maturation of cultured CGD MoMacs. TNFα neutralization also reduced production of IL-1β, IL-6 and CXCL1 by CGD cells though these cytokines played no role in MoMac programming. MoMacs lacking TNFR1 matured more normally in the CGD milieu both *ex vivo* and following adoptive transfer *in vivo*.

**Discussion:**

These data lend mechanistic insights into the utility of TNFα blockade in CGD and to other diseases where such therapy has been shown to be beneficial.

## 1 Introduction

Defects in the production of reactive oxygen species (ROS) by the phagocyte NADPH oxidase Nox2 result in chronic granulomatous disease (CGD), a condition impairing both the antimicrobial capacity of phagocytes and the regulation of the inflammatory response more broadly. Affected patients are highly susceptible to infection with certain pathogens but also display hyperinflammatory responses to non-infectious stimuli (1, 2). Granulomatous inflammation (often sterile), colitis and autoimmunity are frequent and often require immunosuppressive therapy to control (3–5). Murine models mimic the human disease and provide insights into the hyper-inflammatory responses seen in CGD (6–10).

Numerous studies of neutrophils and macrophages from both CGD patients and the murine models demonstrate the over-production of pro-inflammatory mediators (8, 11–13). This and impairments in pro-resolving macrophage programming, including efferocytosis, the ability to clear dying cells and tissue debris (14–16), are hypothesized to contribute significantly to CGD hyperinflammatory responses. Focusing on monocyte-derived macrophages (MoMacs) in a zymosan peritonitis model, we recently characterized the failure of CGD MoMacs to mature in the same manner as WT MoMacs (17). Importantly, using bone marrow chimeras and reciprocal adoptive transfers, it was shown that WT MoMacs failed to mature in the CGD peritoneum whereas CGD MoMacs matured in the WT environment. This study and another demonstrate that factors in the inflammatory milieu in CGD mice prevent this transition, rather than the absence of Nox2 activity being an intrinsic requirement for the gain of pro-resolving functions by macrophages (18).

Using the gp91^phox -/-^ or ^-/y^ murine model of X-linked CGD, we sought to identify factor(s) in the milieu of the inflamed peritoneum of mice deficient in ROS production that inhibit acquisition of pro-resolving characteristics of MoMacs, as an understanding of this derailment might be exploited therapeutically. However, the unabated recruitment of new monocytes from the blood in CGD animals posed significant challenges to identifying determinants of CGD MoMac programming over time (17). A reductionist *ex vivo* approach was adopted using culture of total leukocytes from inflamed peritoneal lavage to recreate the maturation of WT MoMacs over time, and the failure of CGD MoMacs to mature, as observed *in vivo*. Maturation was defined by expression of marker proteins, reduction of pro-inflammatory mediator production and gain of efferocytic capacity, a key function of pro-resolving macrophages. Importantly, this system overcomes the challenges related to continuous CGD MoMac recruitment *in vivo*, retains the contribution of other cell types (particularly neutrophils) to the inflammatory milieu, and allows for easy manipulation of the environment for the identification of key signals. Manipulations of the milieu demonstrated that TNFα signaling via TNFR1, and not other prominent cytokines, was the primary driver thwarting CGD MoMac maturation. As proof of concept, this was then tested *in vivo:* TNFα blocking antibody administered during peritonitis in CGD animals promoted maturation of recruited MoMacs and adoptively transferred MoMacs lacking TNFR1 went on to phenotypically mature in the inflamed CGD peritoneum. Taken together, these findings lend mechanistic insight into TNFα blockade used in CGD and in other chronic diseases where macrophage pro-resolving programming is derailed.

## 2 Methods

### 2.1 Animals

The following mice were obtained from Jackson Laboratories: C57Bl/6 (stock #000664), CD45.1(#002014), gp91phox ^-/-^ and ^-/y^ (#002365), TNFR1^-/-^ (#003242) and bred in-house in a specific pathogen-free facility at National Jewish Health. Both male and female mice were used at 8-12 weeks of age, in accordance with protocols approved by the IACUC.

Experimental mice were treated with 200μg zymosan in PBS (Sigma) via intraperitoneal (i.p.) injection. Mice were euthanized by CO_2_ asphyxiation 20 hours post-zymosan injection for *ex vivo* culture or 72 hours post-zymosan for MoMac phenotyping. TNFα neutralizing antibody (clone XT3.11, BioXCell) or isotype-matched control antibody was delivered by i.p. injection of 300μg per mouse at 20h and 44h post-zymosan. Peritoneal lavage was performed using 8mL lavage buffer (HBSS/10mM HEPES/1mM EDTA).

### 2.2 *Ex vivo* cell culture

Single cell suspensions were made by passing lavage through a 100μm nylon cell strainer. Cells were washed at least once in RPMI culture media (RPMI/10%HI-FBS/100U/mL Penicillin/100U/mL Streptomycin/0.29mg/mL L-glutamine), counted and plated at a constant density of 1x10^6^ total cells/well in 24 well tissue culture treated plates, unless otherwise noted. Neutralizing antibodies or isotype-matched control antibodies (**Table 1**) were added at the time of plating. Some cells were labeled with 10μM Pacific Blue Succinimidyl Ester (PBSE) before culture. Plates were incubated at 37°C with 5% CO_2_.

**Table 1:** Neutralizing antibodies and controls used *in vitro*.

Antibody	Clone	Vendor	Final Concentration
Anti-TNFα	MP6-XT22	BioLegend	5μg/mL
Anti-IL1β	B122	BioXCell	10μg/mL
Anti-IL6	MP5-20F3	BioLegend	5μg/mL
Anti-IFNAR1	MAR1-5A3	BioLegend	20μg/mL
Anti-IFNγ	XMG1.2	BioLegend	10μg/mL
Rat IgG1	HRPN	BioXCell	5μg/mL
Mouse IgG	DV5.1	BioLegend	20μg/mL
Armenian Hamster IgG	polyclonal	BioXCell	10μg/mL
Rat IgG2a	2A3	BioXCell	5μg/mL

Conditioned media was collected after 24 hours in culture and centrifuged to pellet cells prior to treatment of newly plated cells. Proteinase K or DNase (Sigma) were used at 50μg/mL for 25 minutes at 37°C followed by heat inactivation of the proteinase by boiling at 95°C for 5 minutes.

To harvest cells for phenotyping, suspension cells were collected into tubes followed by trypsin treatment of adherent cells at 37°C for 5 minutes. Adherent and suspension cells were pooled for analysis.

### 2.3 Multiplex cytokine analysis

Cell-free culture supernatants or low volume peritoneal lavage fluid was collected at the indicated timepoints and stored at -80°C with protease inhibitor cocktail (Thermo). Cytokine detection and quantitation was performed using the LEGENDPlex assay (BioLegend) according to the manufacturer’s instructions.

### 2.4 Efferocytosis assay

For neutrophil isolation CD45.1 mice were anesthetized using an isoflurane vaporizer and 1μg recombinant mouse CXCL1 (Peprotech) was delivered intranasally in PBS. Bronchoalveolar lavage (BAL) was collected after 3 hours, at which time >97% of recovered leukocytes were neutrophils. BAL cells were labeled with 5μM Carboxyfluorescein succinimidyl ester. After overnight culture ~60% of neutrophils were apoptotic as defined by annexin V staining, performed according to manufacturer’s instructions (BD Bioscience). These were co-incubated for 1 hour with peritoneal lavage cells after 47h in culture at an estimated ratio of 1:1 apoptotic neutrophils: MoMacs before collection for flow cytometry. Engulfing MoMacs were defined as CD64^+^ CFSE^+^ CD45.1^-^ Ly6G^-^ to exclude MoMacs that had bound but not internalized target cells.

### 2.5 Flow cytometry

Cells from lavage or *ex vivo* culture were resuspended in Flow cytometry buffer (HBSS/3% FBS) with anti-CD16/32 antibody to block Fc receptors. Fluorescently-conjugated staining antibodies were added (detailed in **Table 2**) for >30 minutes. Sytox Blue dye (Invitrogen) was added prior to data acquisition to exclude dead cells. For intracellular cytokine staining, plated cells were pre-treated with 5μg/mL Brefeldin A (BioLegend) +/- 100ng/mL LPS re-stimulation 4 hours prior to collection. Cells were stained for surface markers as described then fixed, permeabilized and stained with anti-cytokine antibody using the FoxP3 staining buffer set (eBioscience, Thermo).

**Table 2:** Antibodies for FACS.

Antigen	Conjugate	Clone	Vendor
CD16/32	unconjugated	93	BioLegend
CD54	AF488	YN1/1.7.4	BioLegend
CD80	PerCP Cy5.5	16-10A1	BioLegend
CD206	PE	CO68C2	BioLegend
CD64	PE-Cy7	X54-5/7.1	BioLegend
CD36	APC	HM36	BioLegend
Ly6C	APC-Cy7	HK1.4	BioLegend
F4/80	BV510	BM8	BioLegend
Tim4	BUV395	21H12	BD Biosciences
Ly6G	BV421/BUV395	1A8	BioLegend/BD
B220	BV421/BUV395	RA3-6B2	BioLegend/BD
CD45.1	BV421/BUV395	A20	BioLegend/BD
SiglecF	BV421/BUV395	E50-2440	BD Biosciences
TNFα	APC	MP6-XT22	BioLegend

Flow cytometry data was acquired on an LSRII or LSR Fortessa flow cytometer (BD Bioscience) and analyzed using FlowJo v10 (BD Bioscience). tSNE analysis was performed using FlowJo default settings on concatenated fcs files after gating to select live, single MoMacs representing >2 replicate wells/mice per condition after subsampling to equalize numbers of input MoMacs between samples. “Mature” phenotype was defined as that represented by the majority of untreated WT control MoMacs, as described in individual figure legends.

### 2.6 Statistical analysis

Statistical analysis was performed using Prism software (Graphpad). Details of the statistical tests performed are described in figure legends.

## 3 Results

### 3.1 *Ex vivo* MoMac culture recapitulates *in vivo* differences between CGD and WT monocyte-to-macrophage maturation

**Figure 1:**
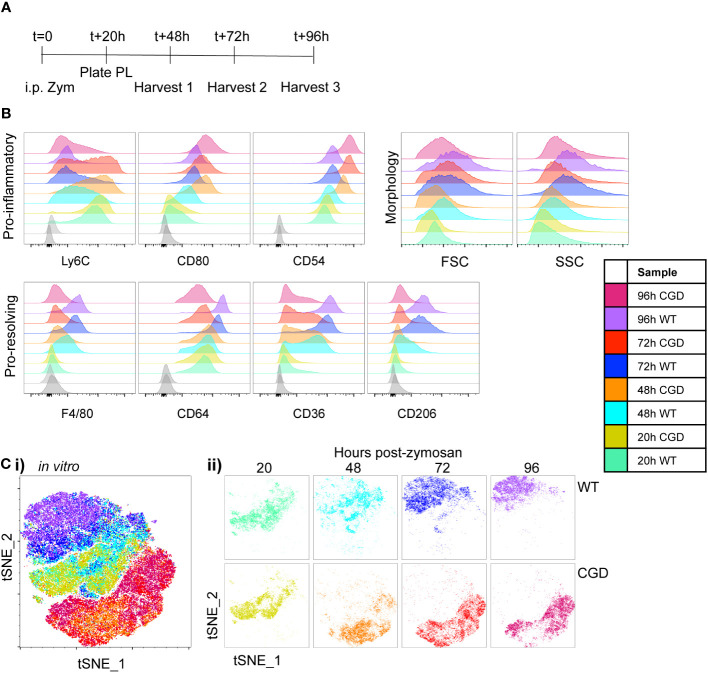
*Ex vivo* MoMac culture recapitulates the *in vivo* failure of CGD monocyte-to-macrophage maturation. Peritoneal lavage (PL) cells were collected from C57BL/6 (WT) or gp91phox^-/-^ (CGD) mice at 20 hours post zymosan and plated for *ex vivo* culture. Changes in MoMac phenotype over time were analyzed by flow cytometry. Data are representative of n=2 independent experiments showing data from n=3 mice/genotype. **(A)** Timeline for *ex vivo* experiments. **(B)** Representative histograms show relative expression by MoMacs of each phenotyping marker used. **(C)** Each parameter shown in part B was used in tSNE analysis of pooled MoMacs after normalizing for MoMac number, shown with each sample overlaid in tSNE space (i) or separated for clarity (ii).

To study MoMac maturation in a controlled environment and identify factors within the CGD milieu that prevent maturation, we developed an *ex vivo* culture system and method to define maturational state by flow cytometry. Total peritoneal lavage cells were collected from WT and CGD mice 20 hours after the onset of zymosan-induced peritonitis and cultured over time (**Figure 1A**). At this timepoint, MoMacs of both genotypes were relatively immature, and phenotypically and transcriptionally similar (17). The composition of cell types placed in culture differed somewhat between genotypes reflecting their distributions in the peritoneal cavity at the time of harvest: a higher proportion of neutrophils was present in the lavage of CGD mice, and a higher percentage of B lymphocytes present in WT mice (**Supplementary Figure 1**). Overall cell numbers declined over time, mostly representing the loss of neutrophils within the first 24 hours after plating, while MoMac numbers were similar between genotypes and declined only slightly over time (**Supplementary Figure 1**). Notably, very few resident peritoneal macrophages were present in lavage at this timepoint for either genotype (Tim4^+^, < 2% of total CD64^+^ cells (19, 20)). The vast majority (>98%) of plated macrophages were Ly6C^+^ MoMacs derived from recruited monocytes in response to zymosan stimulation. In all further analyses MoMacs were identified in peritoneal lavage as live, single cells lacking expression of Ly6G, Siglec F and B220 and expressing CD64 and F4/80.

MoMac maturation over time in culture was investigated via expression of a panel of monocyte/macrophage marker proteins described in the literature. Ly6C is used ubiquitously to describe a population of circulating monocytes in the mouse. Although its molecular function is still unclear, down-regulation of this marker correlates with macrophage maturation (21–23). At 20h post-zymosan, MoMacs from both WT and CGD mice express Ly6C and downregulation was observed over time in both genotypes, although significantly delayed in CGD compared to WT (**Figure 1B**). F4/80 and CD64 are widely used to identify macrophages and were both up-regulated on macrophages compared to circulating monocytes (24). This was observed in our *ex vivo* cultured WT MoMacs but to a lesser extent in CGD MoMacs over time. CD54 (ICAM1) and the costimulatory molecule CD80, markers of activated pro-inflammatory cells (25), were both up-regulated by CGD MoMacs over time in culture (**Figure 1B**; **Supplementary Figure 2**). In contrast pro-resolving macrophages up-regulate expression of CD36 and CD206, scavenger receptors binding lipids and glycoproteins respectively, with important functions in the clearance of apoptotic cells and inflammatory debris (14, 26, 27). These were both steadily up-regulated over time by WT but not CGD MoMacs (**Figure 1B**; **Supplementary Figure 2**). Increases in size and granularity measured by forward and side-scatter (FSC, SSC) are hallmarks of macrophage differentiation from monocytes. In this model, there was a less consistent association between FSC and SSC characteristics and MoMac maturation *ex vivo*, possibly because MoMac morphology can be altered by adhesion to plastic tissue culture plates (**Supplementary Figure 2**). Additional phenotyping markers were also explored (**Supplementary Figure 3**): staining for CD226, CCR2, CD11c and Tim4 was lacking on WT and CGD MoMacs, and expression of CD11b, CD115, CD14, CX_3_CR1, MHCII and MerTK did little to distinguish between genotypes *ex vivo* and were not explored in further analyses.

To aid the visualization of the phenotypic differences described by the marker panel in **Figure 1B**, we used tSNE analysis of pooled MoMac samples after normalizing for differences in cell numbers between timepoints and genotypes. This analysis confirmed the similarity of WT and CGD MoMacs at the time of plating, but showed a strong separation of the two genotypes after the first 24h in culture that persisted over at least 3 days in culture (**Figure 1C**). This separation was initially the result of the up-regulation of CD54 and CD80 by CGD MoMacs and was later strengthened by the down-regulation of Ly6C and up-regulated expression of the pro-resolving markers CD64, CD36 and CD206 by WT cells.

**Figure 2:**
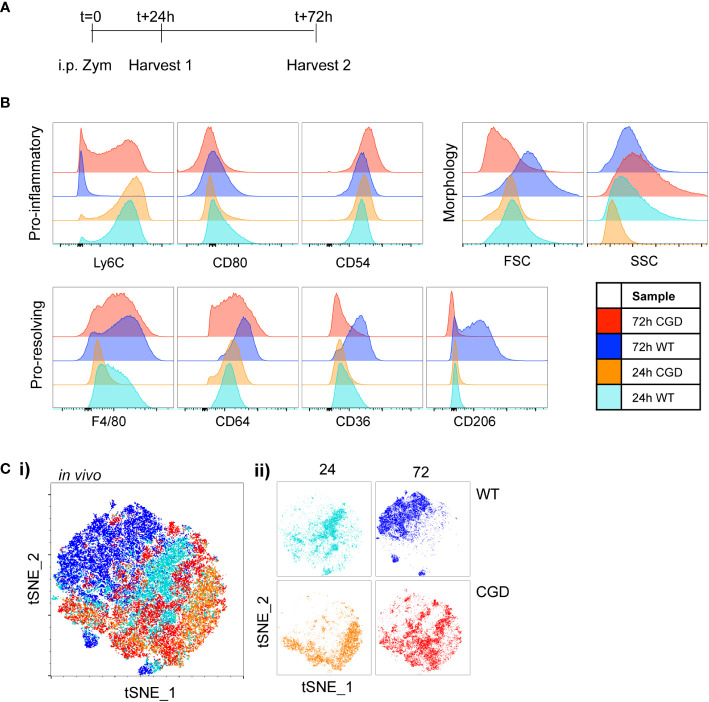
Characterization of *in vivo* MoMac maturation Peritoneal lavage was collected at either 24h or 72h after i.p. zymosan treatment of WT or CGD mice and MoMac phenotype was analyzed by flow cytometry. Representative of n>2 independent experiments with 3 mice/timepoint/genotype. **(A)** Timeline for *in vivo* experiments. **(B)** Representative histograms show relative expression by MoMacs of each phenotyping marker used. **(C)** Each parameter shown in part B was used in tSNE analysis of total pooled MoMacs after normalizing for MoMac number, shown with each sample overlaid in tSNE space (i) or separated for clarity (ii).

To validate the use of this panel *in vivo* we collected samples of peritoneal lavage cells from WT and CGD mice at 24 or 72 hours following intraperitoneal zymosan injection (**Figure 2A**). As expected, WT MoMacs down-regulated expression of Ly6C and up-regulated expression of F4/80, CD64, CD36 and CD206 while CGD MoMacs do not (**Figure 2B**; **Supplementary Figure 4**). Of note, expression of CD54 and CD80 was not detected as strongly *in vivo*, perhaps because this population mostly consists of monocytes only recently recruited from circulation as previously proven by PKH26 labeling and tracking (17). tSNE analysis again showed that 24h MoMacs from WT and CGD mice and 72h CGD MoMacs cluster relatively close together in tSNE space due to their shared expression of an “immature” marker profile whereas 72h WT MoMacs separated into a unique cluster (**Figure 2C**). This divergence represented the loss of Ly6C by WT MoMacs and the concurrent gain of pro-resolving markers particularly CD36 and CD206 whereas the 72h CGD MoMacs continued to resemble the early, pro-inflammatory MoMacs as previously described.

Altogether, these data show that *ex vivo* MoMac maturation, and failure to mature in CGD, leads to similar phenotypic changes to those observed *in vivo* as described by the expression of well-defined monocyte/macrophage proteins. These data demonstrate that the culture of total peritoneal cells is sufficient to substantially recreate the different environments that influence MoMac maturation in the peritoneal cavity and to allow identification of key factors preventing MoMac maturation in CGD.

### 3.2 CGD cells secrete factors that inhibit the maturation of co-cultured MoMacs

**Figure 3:**
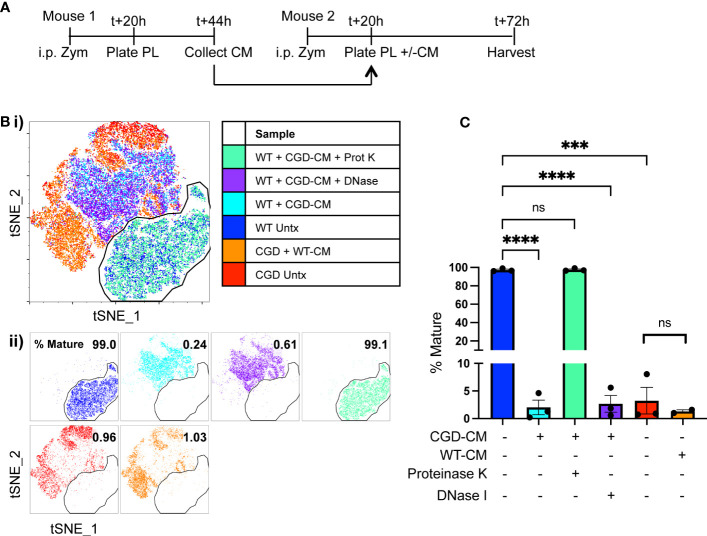
Soluble protein factors released by CGD cells inhibit MoMac maturation *ex vivo.* Conditioned media (CM) was generated by WT or CGD peritoneal lavage cells and added to freshly harvested WT or CGD PL cells as indicated. CGD conditioned media was treated with either proteinase K and boiling, or DNase I prior to addition to test cultures where indicated. **(A)** Experimental timeline. **(B)** tSNE was performed on pooled MoMacs after normalizing for cell numbers. Overlaid (i) and separated samples (ii). Numbers indicate the percentage of MoMacs in each sample falling into the indicated “mature” gate, representing >95% of untreated WT control MoMacs (Blue). **(C)** Summarized data from n=3 experiments showing % mature as defined in part **(B)**, error bars indicate mean ± SEM *** p<0.0005, **** p<0.0001; ns, non-significant by ANOVA.

We hypothesized that the critical cues are secreted, soluble factors produced by lavage cells and hence should accumulate in culture media over time. To this end, conditioned media was collected from plated WT or CGD lavage cells after 24h in culture and this was used to treat a second set of plated lavage cells of either genotype. Treatment of WT MoMacs with CGD-conditioned media prevented the up-regulation of F4/80, CD64, CD36 and CD206, and induced up-regulation of CD54 (**Figures 3A, B**; **Supplementary Figure 5**), indicating that normal maturation was inhibited and MoMacs instead showed pro-inflammatory activation. Treatment of CGD MoMacs with WT-conditioned media had no effect on their phenotype, suggesting that there are no soluble pro-maturing signals produced by WT cells that are able to overcome the pro-inflammatory programming of CGD cells. This was quantified using tSNE analysis of pooled MoMacs. The mature population was defined using the untreated WT MoMac population and a gate was drawn to quantify the percentage of cells from each sample that clustered together with the mature cells (**Figures 3B, C**). This analysis clearly demonstrates that treatment of WT MoMacs with CGD conditioned media completely prevented their normal maturation, while WT conditioned media had no effect on CGD MoMac phenotype. This suggested the production of inhibitory factors by CGD cells in culture that act upon both CGD and WT MoMacs. Notably, treatment with proteinase and boiling eliminated the inhibitory effect of CGD conditioned media, suggesting that one or more protein factors were responsible for inhibition of maturation (**Figure 3C**).

### 3.3 Heightened production of TNFα by CGD myeloid cells inhibits MoMac maturation

**Figure 4:**
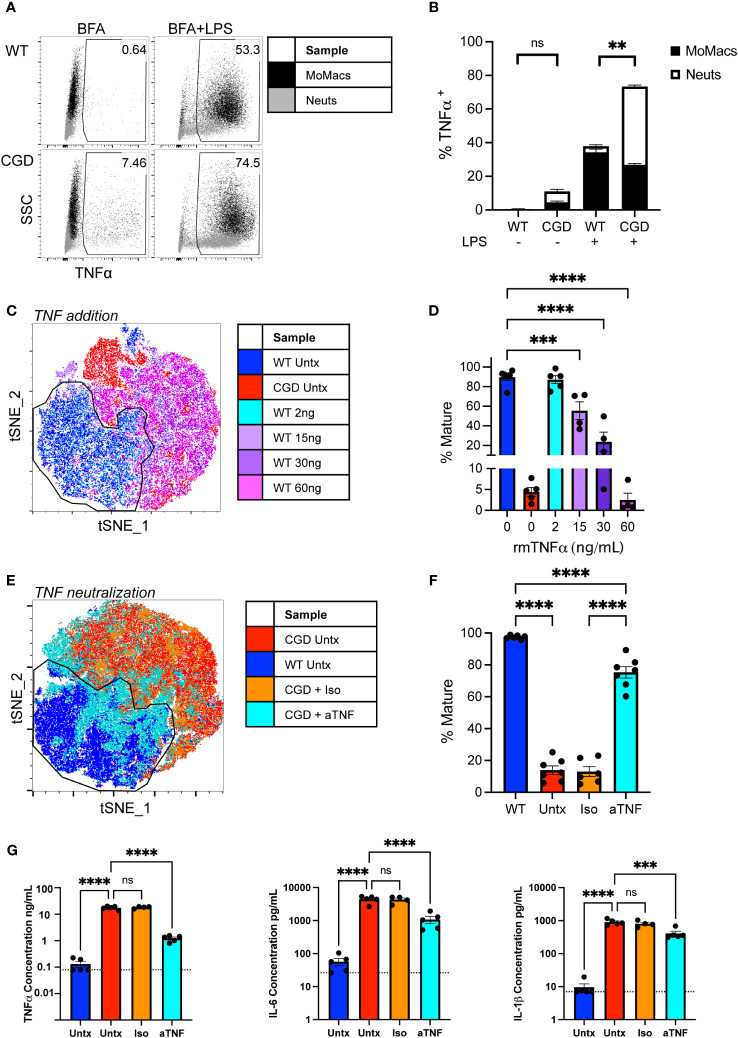
Heightened production of TNFα by CGD myeloid cells inhibits MoMac maturation. **(A)** WT or CGD peritoneal lavage (PL) cells were re-stimulated for 4 hours with brefeldin A (BFA) +/- LPS after 20h *ex vivo*. Intracellular cytokine staining was performed to identify TNFα-expressing cells (x-axis). Data shown represent the percentage of total cultured cells expressing TNFα. **(B)** TNFα-expressing MoMacs or Neutrophils (Neuts) as a percentage of total plated cells as in part **(A)** Data from n=3 experiments, bars indicate mean + SEM. **p<0.01 for both the comparison of TNFα^+^ MoMacs and Neuts between WT and CGD; ns, non-significant by 2-way ANOVA. **(C)** WT PL cells were treated with recombinant murine TNFα protein at the indicated doses for 48 hours. tSNE analysis was performed on pooled MoMacs. Indicated cluster represents >90% of WT control MoMacs. **(D)** Summarized data from >4 independent experiments showing % Mature cells (mean ± SEM) as defined in part C *** p<0.0005, ****p<0.0001 by ANOVA. **(E)** CGD PL cells were plated with TNFα neutralizing antibody (aTNF) or isotype control (Iso) for 48 hours. tSNE analysis was performed on pooled MoMacs. Indicated cluster represents >95% of WT control MoMacs. **(F)** Summarized data from >6 independent experiments showing % Mature cells as defined in part E (mean ± SEM) **** p<0.001 by ANOVA. **(G)** Culture supernatants from 3 independent experiments were analyzed with a multiplex cytokine array after 48h in culture. Dashed line indicates assay lower limit of detection, error bars indicate mean ± SEM, *** p<0.0005, **** p<0.001; ns, non-significant by ANOVA.

CGD cells are known to overproduce a number of pro-inflammatory cytokines in response to PAMP stimulation that contribute substantially to the heightened inflammatory milieu in CGD mice. Of these, TNFα is considered a master regulator of pro-inflammatory cytokine production driving NF-κB and AP-1 activation and downstream pro-inflammatory gene transcription (28), and myeloid cells from CGD patients and animals show both heightened and prolonged production of TNFα in response to fungal particles (13, 29, 30). Elevated concentrations of TNFα were detected in conditioned media from CGD cultures after 48h compared to WT (<130 vs 17,573 ± 2,516 pg/mL). To determine its role in our *ex vivo* model, and to establish the cellular source of TNFα, intracellular cytokine staining was performed after 24h in culture. It was found that both CGD neutrophils and macrophages continue to produce TNFα over time with detectable amounts produced spontaneously between 20 and 24h in the presence of Brefeldin A. Additionally, a greater proportion of CGD cells remained responsive to re-stimulation (**Figures 4A, B**), producing abundant TNFα in response to brief LPS stimulation.

Treatment of WT cells with increasing concentrations of recombinant TNFα significantly inhibited the maturation of WT MoMacs, most noticeably maintaining Ly6C expression, inducing CD54 expression and preventing the expression of CD36 and CD206 (**Figures 4C, D**; **Supplementary Figure 6A**). Conversely, neutralization of TNFα with blocking antibody was sufficient to restore expression of F4/80, CD64, CD36 and CD206 by CGD MoMacs, so that they more closely resembled WT MoMacs than CGD MoMacs treated with control antibody (**Figures 4E, F**; **Supplementary Figure 6B**). Neither treatment with exogenous TNF or neutralizing antibody significantly affected the survival of cultured leukocytes (**Supplementary Figure 7**).

TNFα neutralization significantly decreased the production of other pro-inflammatory cytokines IL-6 and IL-1β and possibly TNFα itself (**Figure 4G**) and the neutrophil chemoattractant CXCL1 (**Supplementary Figure 8**) suggesting that these are regulated down-stream of TNFα signaling. Concentrations of IFNγ, GM-CSF and IL-10 were also found to be elevated in CGD cultures relative to WT but were unaffected by TNFα neutralization (**Supplementary Figure 8**). Expression of these seems to be regulated independently of TNFα and were not important determinants of MoMac maturation in this model system. Antibody-mediated blockade of IL-1β, IL-6, IFNγ, or type I interferon signaling alone had no effect on CGD MoMac maturation (**Supplementary Figure 9**). Combination blockade of TNFα and IL-1β simultaneously had no additional effect beyond that seen with TNFα neutralization alone (data not shown). These data collectively suggest that TNFα is the predominant driver of pro-inflammatory MoMac programming in the CGD environment promoting both the maintenance of immature phenotype and pro-inflammatory cytokine production.

### 3.4 TNFα neutralization partially restores *in vivo* MoMac maturation

**Figure 5:**
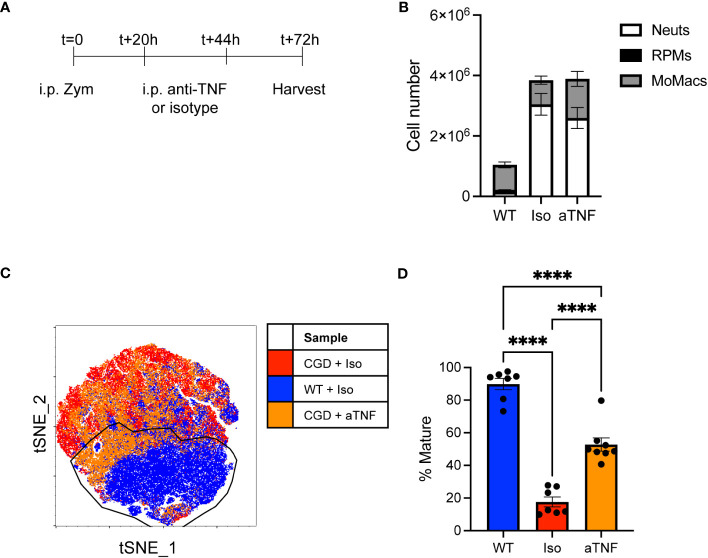
*in vivo* TNFα neutralization improves CGD MoMac maturation. CGD mice were treated with two doses of anti-TNFα neutralizing antibody and peritoneal lavage was collected at 72h. **(A)** Timeline of neutralizing antibody treatment. **(B)** Numbers of cells collected. Representative of n>7 mice per group over 2 experiments, error bars indicate mean ± SEM. **(C)** tSNE analysis of pooled MoMacs from peritoneal lavage at 72h post-zymosan. Indicated cluster represents >70% of WT MoMacs. **(D)** Summarized data from n>7 mice per group over 2 experiments showing % mature as described in part C (mean ± SEM). **** p<0.0001 by ANOVA.

To assess the role of TNFα *in vivo* we delivered neutralizing antibody to CGD mice at 20 and 44 hours after i.p. zymosan treatment and collected peritoneal lavage to assess MoMac phenotype at 72h post-zymosan (**Figure 5A**). Cell numbers or composition in lavage were not significantly altered by anti-TNFα treatment (**Figure 5B**) but the population of lavagable MoMacs differed between anti-TNFα or isotype treated CGD mice. Greater heterogeneity exists in the WT MoMac population *in vivo* as monocytes continue to be recruited into the peritoneal cavity, albeit in low numbers, and so mature cells were defined in tSNE analysis using the Ly6C^lo^ CD64^hi^ CD206^+^ CD36^+^ population from WT lavage for comparison to CGD MoMacs. Approximately half of MoMacs from TNFα-neutralized CGD mice adopted a phenotype that more closely resembled WT MoMacs (**Figures 5C, D**) showing downregulation of CD54 and Ly6C, along with upregulation of CD36 and CD206, although not to quite the extent of WT MoMacs, and upregulation of CD64 above that seen in the WT MoMacs (**Supplementary Figure 10**).

This observation supports our *ex vivo* data demonstrating a key role of TNFα in the impaired maturation of MoMacs in CGD mice, although it also suggests that additional factors likely exist in the more complex environment of the peritoneal cavity.

### 3.5 Mature MoMacs demonstrate improved efferocytic capacity

**Figure 6:**
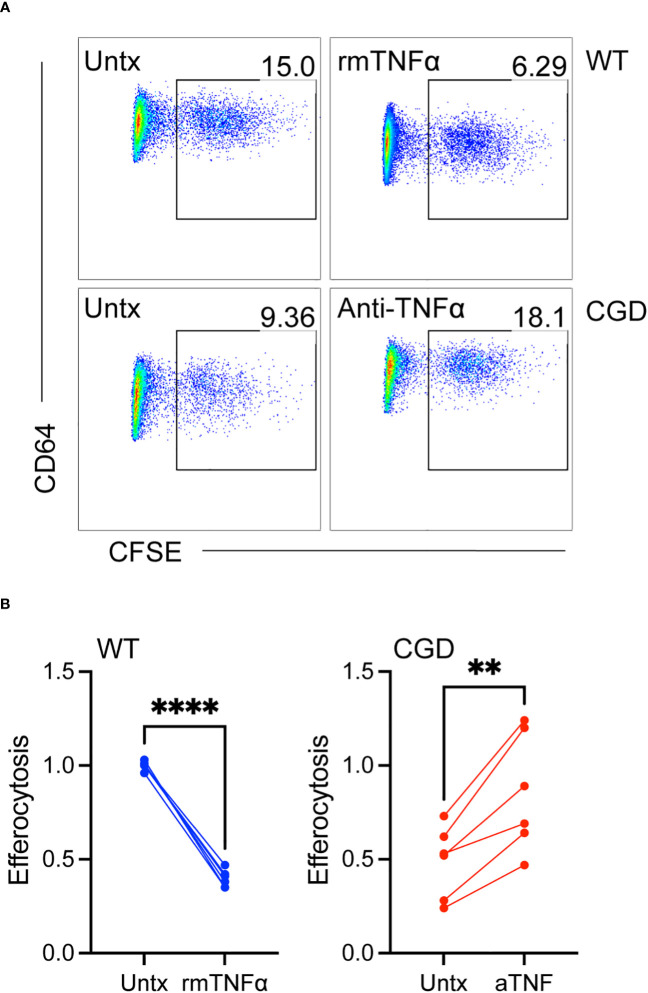
TNFα-mediated inhibition of maturation results in impaired efferocytosis. WT or CGD PL cells were cultured *ex vivo* in the presence or absence of recombinant TNFα or anti-TNFα neutralizing antibody respectively for 47h. CFSE-labeled apoptotic neutrophils were added for 1 hour and the percentage of MoMacs engulfing apoptotic cells was quantified by flow cytometry. **(A)** Representative FACS plots showing the percentage of engulfing macrophages, and **(B)** Data from 2 experiments with n=2-3 mice/genotype, each condition normalized to the untreated WT condition within each experiment. Paired t-tests were used to assess differences with treatment ** denotes p<0.01, **** p<0.0001.

Since TNFα neutralization was able to promote phenotypic maturation we next tested whether it was able to restore efferocytosis, an important pro-resolving function of mature macrophages. Efferocytosis has been shown to be deficient in CGD macrophages (15, 16, 31, 32) and to be inhibited in other macrophage systems by TNFα (33–35). As shown in **Figure 6**, 20h WT MoMacs allowed to mature for 2 days *ex vivo* or cultured with additional exogenous TNFα to inhibit their maturation were tested for their ability to efferocytose apoptotic neutrophils. TNFα significantly reduced the ability of these cells take up apoptotic neutrophils. This appeared to be due to TNFα-induced programming rather than acute effects on efferocytosis that have been described with short-term stimulation (36, 37) as stimulation with TNFα concurrent with apoptotic cell introduction had no effect on uptake (not shown). Conversely, 20h CGD MoMacs cultured for the same time remained immature in phenotype and significantly poorer in their capacity to take up apoptotic neutrophils. Note that the data shown were normalized to the average engulfment of untreated WT MoMacs within each experiment to account for heterogeneity in the preparation of apoptotic neutrophils. As hypothesized, neutralization of TNFα with antibody enhanced both their maturation and efferocytic capacity. Hence, the neutralization of TNFα permitted both the phenotypic maturation of CGD MoMacs and improvements in efferocytic function, a key behavior of mature pro-resolving macrophages.

### 3.6 TNFα -TNFR1 signaling maintains pro-inflammatory MoMacs

**Figure 7:**
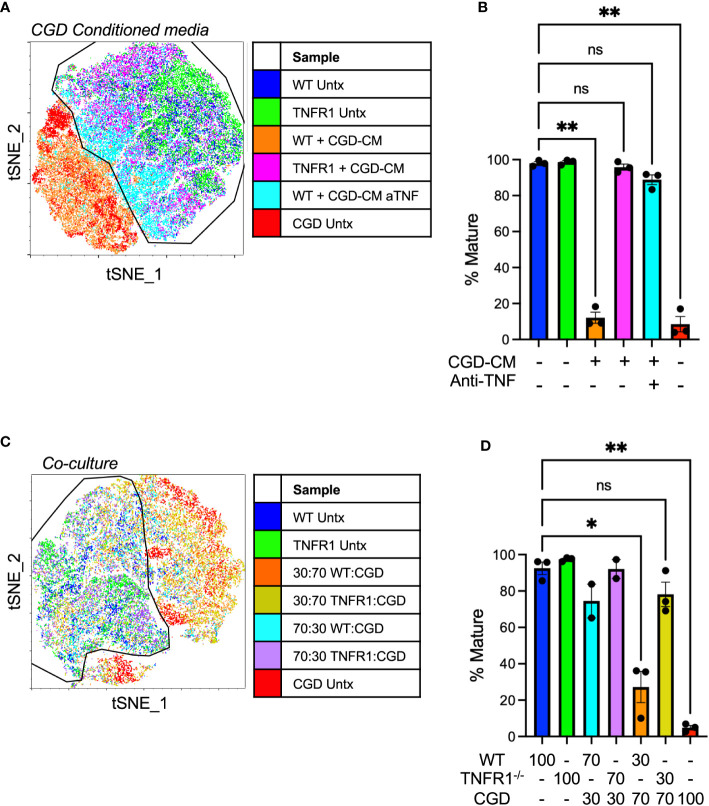
TNFα-TNFR1 signaling maintains pro-inflammatory MoMacs. **(A)** WT or TNFR1^-/-^ 20h post-zymosan lavage cells were treated with CGD conditioned media generated in the presence or absence of TNFα neutralizing antibody. Indicated cluster represents >95% of WT control cells. **(B)** Summarized data from n=3 independent experiments showing % mature MoMacs as defined in part A (mean ± SEM). ** p<0.01; ns, non-significant by ANOVA. **(C)** 20h post-zymosan lavage cells were plated in co-cultures of either 70:30 or 30:70 ratios of CGD : WT or CGD : TNFR1^-/-^ for 48 hours. tSNE analysis performed on equal numbers of total MoMacs per sample after gating to exclude the CGD fraction. Indicated cluster represents >90% of WT control cells. **(D)** Summarized data from >2 independent experiments showing % mature as defined in part C (mean ± SEM). ANOVA analysis used to identify differences * denotes p<0.05, ** p<0.01; ns, non-significant.

WT and CGD MoMacs express both receptors for TNFα: TNFR1 and TNFR2 (**Supplementary Figure 11**). While surface staining for both receptors was significantly lower on CGD MoMacs at the time of plating, culture induced up-regulation of both receptors in both genotypes such that receptor expression was similar after 48h *ex vivo*. Soluble TNFα activates TNFR1 signaling more efficiently than TNFR2 so we hypothesized that TNFR1-deficient MoMacs would be less susceptible to the pro-inflammatory environment created by CGD PL cells. In the presence of conditioned media from CGD PL cells, TNFR1^-/-^ MoMac maturation was largely unaffected while WT MoMac maturation was inhibited as previously observed (**Figures 7A, B**) Confirming the role of TNFα in this setting, neutralization of TNFα during conditioned media generation was sufficient to mostly restore the maturation of WT MoMacs such that they largely resembled the treated TNFR1^-/-^ MoMacs (**Supplementary Figure 12**).

In direct co-culture experiments WT or TNFR1^-/-^ cells were plated with increasing proportions of CGD lavage cells (70:30 or 30:70 WT or TNFR1^-/-^: CGD) maintaining a constant cell density. PBSE labeling was used to distinguish cells of different genotypes such that the effect of co-culture specifically on the WT or TNFR1^-/-^ MoMacs could be determined. Replicate experiments using different labeling protocols confirmed that PBSE had no effect on the observed phenotype or tSNE clustering. TNFR1^-/-^ MoMacs clustered with untreated mature WT cells while the maturation of TNFR1-sufficient WT MoMacs was inhibited (**Figures 7C, D**; **Supplementary Figure 12**) Together these data confirm that soluble TNFα, detected via TNFR1 signaling, maintains the pro-inflammatory phenotype of MoMacs in the presence of CGD myeloid cells and prevents the acquisition of a pro-resolving macrophage phenotype. Importantly, these observations are independent of any potential effects of antibody-Fc receptor binding by MoMacs previously suggested (38, 39).

### 3.7 TNFR1^-/-^ MoMacs are less responsive to the inflammatory milieu in CGD mice

**Figure 8:**
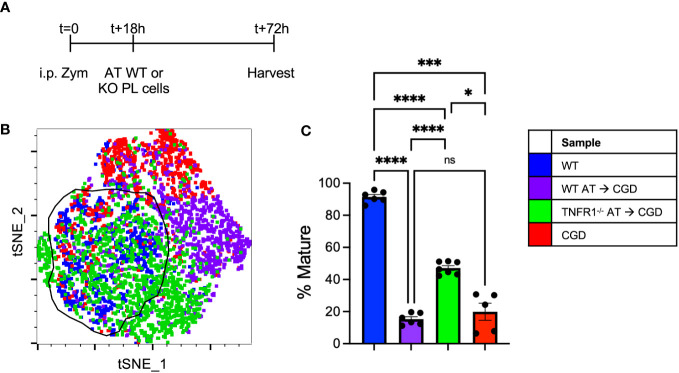
Adoptively transferred TNFR1^-/-^ MoMacs exhibit enhanced maturation relative to WT MoMacs in the CGD peritoneum. WT or TNFR1^-/-^ peritoneal lavage cells were adoptively transferred into inflamed CGD recipient mice. **(A)** Timeline of adoptive transfer (AT) experiments. **(B)** tSNE analysis of pooled MoMacs from peritoneal lavage at 72h post-zymosan. Indicated cluster represents >88% of WT MoMacs. **(C)** Summarized data from n=6-7 mice per group from 2 experiments showing % mature as described in part **(B)** (mean ± SEM). ANOVA analysis was used to identify differences: ns non-significant, * denotes p<0.05, *** p<0.001, **** p<0.0001.

To further demonstrate the necessity of TNFα:TNFR1 signaling *in vivo*, adoptive transfer experiments were performed where WT or TNFR1^-/-^ peritoneal lavage cells collected at 18h post-zymosan were transplanted into the peritonea of inflamed CGD recipient mice (**Figure 8A**). The maturation of transferred MoMacs was assessed at 72h post-zymosan (54h after adoptive transfer). As in the *ex vivo* experiments, adoptive transfer of WT MoMacs into CGD recipients prevented their normal maturation, induced expression of CD54, prevented down-regulation of Ly6C, and up-regulation of CD64, CD36, and CD206. (**Figures 8B, C**; **Supplementary Figure 13**). Adoptively transferred TNFR1^-/-^ MoMacs were not prevented from maturing and underwent down-regulation of Ly6C and up-regulation of CD64, CD36, CD206 and MHCII (**Supplementary Figure 13**). Using tSNE analysis, the phenotype of TNFR1^-/-^ MoMacs showed significant overlap with that of mature MoMacs from control WT mice treated with zymosan alone. These observations support the conclusion of our *ex vivo* experiments that TNFα: TNFR1 signaling is critical in maintaining the pro-inflammatory phenotype of MoMacs in CGD, and that blockade of this signaling permits MoMac maturation.

## 4 Discussion

Macrophages have essential roles in maintaining and restoring tissue homeostasis, and the induction of immune responses. While resident macrophages often fulfill these functions, many insults to tissues call in additional monocytes from circulation that then mature into macrophages (MoMacs). MoMacs are highly plastic cells, expressing a diverse array of receptors for pathogen- or damage-associated molecular patterns (PAMPs, DAMPs), cytokines, and growth factors that make them highly sensitive to cues from pathogens, damaged or activated cells within the injured tissues into which they are recruited (40–43). As such, MoMac programming often shifts during inflammation from pro-inflammatory to pro-resolving as tissues return to homeostasis (21, 23, 44, 45). Our understanding of the key signals for this shift, how it might be derailed in chronic inflammation, and the potential crosstalk between multiple cell types and signals present in the inflammatory environment is incomplete. Improving our understanding of the molecular cues that drive appropriate monocyte/macrophage adaptation to their environment could potentially yield targets and mechanistic insights for future therapeutic intervention in CGD as well as other chronic inflammatory diseases.

In spite of their Nox2 deficiency, CGD monocytes retain the ability to become pro-resolving MoMacs (17, 18) but the CGD environment, dominated by pro-inflammatory signals, appears to prevent this normal progression. Our *ex vivo* culture system demonstrated successful recapitulation of important features of CGD and WT MoMac programming seen *in vivo* while allowing us to control and manipulate the surrounding milieu to investigate critical inhibitory signals. Of the many inflammatory mediators characterizing CGD inflammation (7, 8, 29), TNFα seemed a likely candidate for promoting proinflammatory MoMac programming. TNFα is produced in excess and over a sustained period by both CGD neutrophils and MoMacs following zymosan stimulation (13, 30) with high concentrations accumulating both in the mouse peritoneal cavity and in culture media after plating. As shown, TNFα supplied exogenously, and more importantly, in conditioned media generated by CGD cells signaled through TNFR1 to inhibit pro-resolving programming by Nox2-sufficient MoMacs. This indicates that intrinsic Nox2 activity does not ultimately determine the responsiveness of MoMacs to this signal. Conversely, the blockade of TNFα signaling, either via antibody neutralization or through receptor deficiency, was sufficient to reduce the expression of pro-inflammatory surface markers and promote the gain of a pro-resolving phenotype both *ex vivo* and in CGD mice *in vivo*. Other cytokine candidates were also tested for their contribution to CGD MoMac programming. Neutralization of IL-6 and IL-1β were shown to have no effect on programming. Similarly, blockade of IFNAR1 had no effect. Although there is a signature for IFNγ stimulation in CGD macrophages (17, 18) and significant levels of this cytokine were produced by CGD cells, IFNγ neutralization did not result in WT programming of the MoMacs. Of note, antagonizing TNFα signaling, in addition to significantly normalizing MoMac maturation phenotype also had anti-inflammatory consequences with decreased production of several cytokines and enhanced efferocytic capability.

Further, in the peritonitis model the study of CGD MoMac maturation *in vivo* is confounded by the constant, ongoing recruitment of immature monocytes from circulation. Unlike WT MoMacs which remain in the peritoneal cavity while maturing, CGD MoMacs also have an extremely short dwell time in the peritoneal cavity (estimated half-life of approximately 6 hours over the first 72 hours following zymosan), and are found accumulating at the diaphragm, many sequestered into pyogranulomata (17). TNFα neutralization did not seem to impact CGD MoMac turnover in PKH26 dye labeling experiments (not shown), likely because multiple redundant factors are involved in the ongoing recruitment of monocytes to the peritoneal cavity. This is perhaps why we observed only partial effects of TNFα neutralization on maturation, highlighting one of the major benefits of our *ex vivo* approach. While 2 doses of anti-TNFα had little obvious effect on pyogranuloma formation (data not shown) the role of TNFα in granuloma formation is well-supported (46, 47).

While both CGD neutrophils and MoMacs themselves contribute to TNFα production, CGD neutrophils being more numerous both *ex vivo* and especially *in vivo* likely represent the greater source (30). We speculate that Nox2 activity in these cells normally limits their production of TNFα, with Nox2-derived ROS potentially acting through several mechanisms suggested in the literature. There is evidence that intracellular ROS suppress NF-κB activity (48–50) which would both reduce the response to TNFα: TNFR1 signaling and the production of TNFα downstream of NF-κB activation. The heightened production of TNFα, IL-1, G-CSF and CXCL1 drives further recruitment of neutrophils. Nox2-generated ROS play important roles in the induction of neutrophil death, both apoptosis (51, 52) and NETosis (53, 54), and in the generation of signals for clearance (55). Hence, heightened recruitment and delays in CGD neutrophil death and clearance provide the opportunity for prolonged TNFα production. Many, or all, of these processes likely act simultaneously in Nox2-sufficiency to reduce active TNFα concentrations in the inflammatory milieu, permitting the eventual resolution of inflammation.

Excess TNFα production is not unique to CGD. TNFα-neutralizing therapies are effective in rheumatoid arthritis joints as well as many other chronic inflammatory diseases. Limited trials of TNFα inhibitors in CGD patients have shown effectiveness in the treatment of arthritis (56), bowel fistulas (57) and reducing the symptoms of interstitial bowel disease, a frequent inflammatory complication in this population (5). Unfortunately, use of these agents must be carefully monitored due to increased risk of infection (58, 59). In a recent series, 14 CGD patients with refractory inflammation involving the GI, pulmonary, cutaneous and/or genitourinary systems were treated with anti-TNFα therapy for a median duration of 1.9 years (5). Infections occurred in half with a median time to first infection of 285 days (range 57-974 days) from start of therapy despite standard antimicrobials. All responded to additional anti-infectious treatment. Therapeutic benefit of anti-TNFα treatment was seen in 11/14 (78.6%) of patients, most of whom went on to successful hematopoietic stem cell transplantation.

Our data support others demonstrating an effect of TNFα blockers on promoting the “alternative activation” of patient macrophages (60, 61). Mature programming would reduce the output of inflammatory mediators and is likely to restore the efficient anti-inflammatory clearance of apoptotic or aged neutrophils and other debris, shown to be deficient in CGD (15, 16, 31). In the future, the development of more sophisticated, targeted TNFα neutralizing therapies may permit their use in immunocompromised individuals (62, 63). Alternatively, our data suggest that targeting TNFR1 on MoMacs specifically might be sufficient to promote resolution of chronic inflammation while preserving advantageous TNFα signaling in neutrophils necessary for immune defense. Further investigation will be required to determine which CGD patient factors such as genotype, residual ROS production, concomitant treatment (e.g., IFNγ) and inflammatory complications are best suited for TNFα blockade and whether any of these mechanisms above can be exploited therapeutically in CGD and other chronic inflammatory conditions.

## Data availability statement

The original contributions presented in the study are included in the article/**Supplementary Material**. Further inquiries can be directed to the corresponding author.

## Ethics statement

The animal study was approved by Institutional Animal Care and Use Committee. The study was conducted in accordance with the local legislation and institutional requirements.

## Author contributions

SG: Conceptualization, Formal analysis, Investigation, Methodology, Visualization, Writing – original draft, Writing – review & editing. KH: Formal analysis, Investigation, Methodology, Writing – review & editing. ER: Resources, Writing – review & editing. PH: Funding acquisition, Supervision, Writing – review & editing. DB: Conceptualization, Formal analysis, Funding acquisition, Supervision, Writing – original draft, Writing – review & editing.

## References

[1] Marciano BE Spalding C Fitzgerald A Mann D Brown T Osgood S. Common severe infections in chronic granulomatous disease. Clin Infect Dis (2015) 60(8):1176–83. doi: 10.1093/cid/ciu1154 PMC4400412 25537876

[2] Henrickson SE Jongco AM Thomsen KF Garabedian EK Thomsen IP. Noninfectious Manifestations and Complications of Chronic Granulomatous Disease. J Pediatr Infect Dis Soc (2018) 7(suppl_1):S18–24. doi: 10.1093/jpids/piy014 PMC5946858 29746679

[3] Dinauer MC. Inflammatory consequences of inherited disorders affecting neutrophil function. Blood (2019) 133(20):2130–9. doi: 10.1182/blood-2018-11-844563 PMC6524563 30898864

[4] Schappi MG Jaquet V Belli DC Krause KH. Hyperinflammation in chronic granulomatous disease and anti-inflammatory role of the phagocyte NADPH oxidase. Semin Immunopathol (2008) 30(3):255–71. doi: 10.1007/s00281-008-0119-2 18509648

[5] Conrad A Neven B Mahlaoui N Suarez F Sokol H Ruemmele FM. Infections in patients with chronic granulomatous disease treated with tumor necrosis factor alpha blockers for inflammatory complications. J Clin Immunol (2021) 41(1):185–93. doi: 10.1007/s10875-020-00901-8 33150502

[6] Morgenstern DE Gifford MA Li LL Doerschuk CM Dinauer MC. Absence of respiratory burst in X-linked chronic granulomatous disease mice leads to abnormalities in both host defense and inflammatory response to Aspergillus fumigatus. J Exp Med (1997) 185(2):207–18. doi: 10.1084/jem.185.2.207 PMC2196125 9016870

[7] Whitmore LC Goss KL Newell EA Hilkin BM Hook JS Moreland JG. NOX2 protects against progressive lung injury and multiple organ dysfunction syndrome. Am J Physiol Lung Cell Mol Physiol (2014) 307(1):L71–82. doi: 10.1152/ajplung.00054.2014 PMC4080282 24793165

[8] Bhattacharya S Idol RA Yang W Rojas Marquez JD Li Y Huang G. Macrophage NOX2 NADPH oxidase maintains alveolar homeostasis in mice. Blood (2022) 139(19):2855–70. doi: 10.1182/blood.2021015365 PMC9101249 35357446

[9] Liao YC Wu SY Huang YF Lo PC Chan TY Chen CA. NOX2-deficient neutrophils facilitate joint inflammation through higher pro-inflammatory and weakened immune checkpoint activities. Front Immunol (2021) 12:743030. doi: 10.3389/fimmu.2021.743030 34557202 PMC8452958

[10] Jackson SH Gallin JI Holland SM. The p47phox mouse knock-out model of chronic granulomatous disease. J Exp Med (1995) 182(3):751–8. doi: 10.1084/jem.182.3.751 PMC2192153 7650482

[11] Bylund J MacDonald KL Brown KL Mydel P Collins LV Hancock RE. Enhanced inflammatory responses of chronic granulomatous disease leukocytes involve ROS-independent activation of NF-kappa B. Eur J Immunol (2007) 37(4):1087–96. doi: 10.1002/eji.200636651 17330823

[12] Brown KL Bylund J MacDonald KL Song-Zhao GX Elliott MR Falsafi R. ROS-deficient monocytes have aberrant gene expression that correlates with inflammatory disorders of chronic granulomatous disease. Clin Immunol (2008) 129(1):90–102. doi: 10.1016/j.clim.2008.06.005 18676204

[13] Yoo DG Paracatu LC Xu E Lin X Dinauer MC. NADPH oxidase limits collaborative pattern-recognition receptor signaling to regulate neutrophil cytokine production in response to fungal pathogen-associated molecular patterns. J Immunol (2021) 207(3):923–37. doi: 10.4049/jimmunol.2001298 PMC8425286 34301842

[14] Fernandez-Boyanapalli RF Frasch SC McPhillips K Vandivier RW Harry BL Riches DW. Impaired apoptotic cell clearance in CGD due to altered macrophage programming is reversed by phosphatidylserine-dependent production of IL-4. Blood (2009) 113(9):2047–55. doi: 10.1182/blood-2008-05-160564 PMC2651016 18952895

[15] Sanmun D Witasp E Jitkaew S Tyurina YY Kagan VE Ahlin A. Involvement of a functional NADPH oxidase in neutrophils and macrophages during programmed cell clearance: implications for chronic granulomatous disease. Am J Physiol Cell Physiol (2009) 297(3):C621–31. doi: 10.1152/ajpcell.00651.2008 19570889

[16] Bagaitkar J Huang J Zeng MY Pech NK Monlish DA Perez-Zapata LJ. NADPH oxidase activation regulates apoptotic neutrophil clearance by murine macrophages. Blood (2018) 131(21):2367–78. doi: 10.1182/blood-2017-09-809004 PMC5969376 29618478

[17] Gibbings SL Haist KC Nick H Frasch SC Glass TH Vestal B. Heightened turnover and failed maturation of monocyte-derived macrophages in murine chronic granulomatous disease. Blood (2022) 139(11):1707–21. doi: 10.1182/blood.2021011798 PMC8931516 34699591

[18] Meda Spaccamela V Valencia RG Pastukhov O Duppenthaler A Dettmer MS Erb J. High levels of IL-18 and IFN-gamma in chronically inflamed tissue in chronic granulomatous disease. Front Immunol (2019) 10:2236. doi: 10.3389/fimmu.2019.02236 31681257 PMC6813411

[19] Ginhoux F Guilliams M. Tissue-resident macrophage ontogeny and homeostasis. Immunity (2016) 44(3):439–49. doi: 10.1016/j.immuni.2016.02.024 26982352

[20] Zhang N Czepielewski RS Jarjour NN Erlich EC Esaulova E Saunders BT. Expression of factor V by resident macrophages boosts host defense in the peritoneal cavity. J Exp Med (2019) 216(6):1291–300. doi: 10.1084/jem.20182024 PMC6547866 31048328

[21] Dal-Secco D Wang J Zeng Z Kolaczkowska E Wong CH Petri B. A dynamic spectrum of monocytes arising from the in *situ* reprogramming of CCR2+ monocytes at a site of sterile injury. J Exp Med (2015) 212(4):447–56. doi: 10.1084/jem.20141539 PMC4387291 25800956

[22] Varga T Mounier R Gogolak P Poliska S Chazaud B Nagy L. Tissue LyC6- macrophages are generated in the absence of circulating LyC6- monocytes and Nur77 in a model of muscle regeneration. J Immunol (2013) 191(11):5695–701. doi: 10.4049/jimmunol.1301445 24133167

[23] Crane MJ Daley JM van Houtte O Brancato SK Henry WL Jr. Albina JE. The monocyte to macrophage transition in the murine sterile wound. PloS One (2014) 9(1):e86660. doi: 10.1371/journal.pone.0086660 24466192 PMC3899284

[24] Gautier EL Shay T Miller J Greter M Jakubzick C Ivanov S. Gene-expression profiles and transcriptional regulatory pathways that underlie the identity and diversity of mouse tissue macrophages. Nat Immunol (2012) 13(11):1118–28. doi: 10.1038/ni.2419 PMC3558276 23023392

[25] Goebeler M Roth J Kunz M Sorg C. Expression of intercellular adhesion molecule-1 by murine macrophages is up-regulated during differentiation and inflammatory activation. Immunobiology (1993) 188(1-2):159–71. doi: 10.1016/S0171-2985(11)80495-X 8104878

[26] Boada-Romero E Martinez J Heckmann BL Green DR. The clearance of dead cells by efferocytosis. Nat Rev Mol Cell Biol (2020) 21(7):398–414. doi: 10.1038/s41580-020-0232-1 32251387 PMC7392086

[27] Fadok VA Warner ML Bratton DL Henson PM. CD36 is required for phagocytosis of apoptotic cells by human macrophages that use either a phosphatidylserine receptor or the vitronectin receptor (alpha v beta 3). J Immunol (1998) 161(11):6250–7. doi: 10.4049/jimmunol.161.11.6250 9834113

[28] Parameswaran N Patial S. Tumor necrosis factor-alpha signaling in macrophages. Crit Rev Eukaryot Gene Expr (2010) 20(2):87–103. doi: 10.1615/CritRevEukarGeneExpr.v20.i2.10 21133840 PMC3066460

[29] Segal BH Han W Bushey JJ Joo M Bhatti Z Feminella J. NADPH oxidase limits innate immune responses in the lungs in mice. PloS One (2010) 5(3):e9631. doi: 10.1371/journal.pone.0009631 20300512 PMC2838778

[30] Cagnina RE Michels KR Bettina AM Burdick MD ScIndia Y Zhang Z. Neutrophil-derived tumor necrosis factor drives fungal acute lung injury in chronic granulomatous disease. J Infect Dis (2021) 224(7):1225–35. doi: 10.1093/infdis/jiab188 PMC8682762 33822981

[31] Fernandez-Boyanapalli R McPhillips KA Frasch SC Janssen WJ Dinauer MC Riches DW. Impaired phagocytosis of apoptotic cells by macrophages in chronic granulomatous disease is reversed by IFN-gamma in a nitric oxide-dependent manner. J Immunol (2010) 185(7):4030–41. doi: 10.4049/jimmunol.1001778 PMC4346245 20805415

[32] Anjani G Vignesh P Joshi V Shandilya JK Bhattarai D Sharma J. Recent advances in chronic granulomatous disease. Genes Dis (2020) 7(1):84–92. doi: 10.1016/j.gendis.2019.07.010 32181279 PMC7063432

[33] Lee J Lu Y Oshins R West J Moneypenny CG Han K. Alpha 1 antitrypsin-deficient macrophages have impaired efferocytosis of apoptotic neutrophils. Front Immunol (2020) 11:574410. doi: 10.3389/fimmu.2020.574410 33329539 PMC7714766

[34] Lee HN Kundu JK Cha YN Surh YJ. Resolvin D1 stimulates efferocytosis through p50/p50-mediated suppression of tumor necrosis factor-alpha expression. J Cell Sci (2013) 126(Pt 17):4037–47. doi: 10.1242/jcs.131003 23788426

[35] Michlewska S Dransfield I Megson IL Rossi AG. Macrophage phagocytosis of apoptotic neutrophils is critically regulated by the opposing actions of pro-inflammatory and anti-inflammatory agents: key role for TNF-alpha. FASEB J (2009) 23(3):844–54. doi: 10.1096/fj.08-121228 18971259

[36] McPhillips K Janssen WJ Ghosh M Byrne A Gardai S Remigio L. TNF-alpha inhibits macrophage clearance of apoptotic cells via cytosolic phospholipase A2 and oxidant-dependent mechanisms. J Immunol (2007) 178(12):8117–26. doi: 10.4049/jimmunol.178.12.8117 17548650

[37] Ren Y Savill J. Proinflammatory cytokines potentiate thrombospondin-mediated phagocytosis of neutrophils undergoing apoptosis. J Immunol (1995) 154(5):2366–74. doi: 10.4049/jimmunol.154.5.2366 7532668

[38] McRae BL Levin AD Wildenberg ME Koelink PJ Bousquet P Mikaelian I. Fc receptor-mediated effector function contributes to the therapeutic response of anti-TNF monoclonal antibodies in a mouse model of inflammatory bowel disease. J Crohns Colitis. (2016) 10(1):69–76. doi: 10.1093/ecco-jcc/jjv179 26429698

[39] Mitoma H Horiuchi T Tsukamoto H Ueda N. Molecular mechanisms of action of anti-TNF-alpha agents - Comparison among therapeutic TNF-alpha antagonists. Cytokine (2018) 101:56–63. doi: 10.1016/j.cyto.2016.08.014 27567553

[40] Sanin DE Ge Y Marinkovic E Kabat AM Castoldi A Caputa G. A common framework of monocyte-derived macrophage activation. Sci Immunol (2022) 7(70):eabl7482. doi: 10.1126/sciimmunol.abl7482 35427180 PMC12362712

[41] Orozco SL Canny SP Hamerman JA. Signals governing monocyte differentiation during inflammation. Curr Opin Immunol (2021) 73:16–24. doi: 10.1016/j.coi.2021.07.007 34411882 PMC8648978

[42] Xue J Schmidt SV Sander J Draffehn A Krebs W Quester I. Transcriptome-based network analysis reveals a spectrum model of human macrophage activation. Immunity (2014) 40(2):274–88. doi: 10.1016/j.immuni.2014.01.006 PMC3991396 24530056

[43] Baek SE Jang EJ Choi JM Choi YW Kim CD. alpha-Iso-cubebene attenuates neointima formation by inhibiting HMGB1-induced monocyte to macrophage differentiation via suppressing ROS production. Int Immunopharmacol (2022) 111:109121. doi: 10.1016/j.intimp.2022.109121 35940074

[44] Okabe Y Medzhitov R. Tissue-specific signals control reversible program of localization and functional polarization of macrophages. Cell (2014) 157(4):832–44. doi: 10.1016/j.cell.2014.04.016 PMC4137874 24792964

[45] Zhu X Meyers A Long D Ingram B Liu T Yoza BK. Frontline Science: Monocytes sequentially rewire metabolism and bioenergetics during an acute inflammatory response. J Leukoc Biol (2019) 105(2):215–28. doi: 10.1002/JLB.3HI0918-373R PMC6466628 30633362

[46] Maltesen HR Nielsen CH Dalboge CS Baslund B. Methylprednisolone prevents tumour necrosis factor-alpha-dependent multinucleated giant cell formation. Rheumatol (Oxford) (2010) 49(11):2037–42. doi: 10.1093/rheumatology/keq213 20634232

[47] Silva D Silva MVD Barros CCO Alexandre PBD Timoteo RP Catarino JS. TNF-alpha blockade impairs in *vitro* tuberculous granuloma formation and down modulate Th1, Th17 and Treg cytokines. PloS One (2018) 13(3):e0194430. doi: 10.1371/journal.pone.0194430 29543912 PMC5854376

[48] Warnatsch A Tsourouktsoglou TD Branzk N Wang Q Reincke S Herbst S. Reactive oxygen species localization programs inflammation to clear microbes of different size. Immunity (2017) 46(3):421–32. doi: 10.1016/j.immuni.2017.02.013 PMC5965455 28314592

[49] Han W Li H Cai J Gleaves LA Polosukhin VV Segal BH. NADPH oxidase limits lipopolysaccharide-induced lung inflammation and injury in mice through reduction-oxidation regulation of NF-kappaB activity. J Immunol (2013) 190(9):4786–94. doi: 10.4049/jimmunol.1201809 PMC3633681 23530143

[50] Trevelin SC Dos Santos CX Ferreira RG de Sa Lima L Silva RL Scavone C. Apocynin and Nox2 regulate NF-kappaB by modifying thioredoxin-1 redox-state. Sci Rep (2016) 6:34581. doi: 10.1038/srep34581 27698473 PMC5048297

[51] Kasahara Y Iwai K Yachie A Ohta K Konno A Seki H. Involvement of reactive oxygen intermediates in spontaneous and CD95 (Fas/APO-1)-mediated apoptosis of neutrophils. Blood (1997) 89(5):1748–53. doi: 10.1182/blood.V89.5.1748 9057659

[52] Coxon A Rieu P Barkalow FJ Askari S Sharpe AH von Andrian UH. A novel role for the beta 2 integrin CD11b/CD18 in neutrophil apoptosis: a homeostatic mechanism in inflammation. Immunity (1996) 5(6):653–66. doi: 10.1016/S1074-7613(00)80278-2 8986723

[53] Bianchi M Brinkmann V Siler U Seger RA Zychlinsky A. Restoration of NET formation by gene therapy in CGD controls aspergillosis. Blood (2009) 114(13):2619–22. doi: 10.1182/blood-2009-05-221606 PMC2756123 19541821

[54] Parker HA Jones HM Kaldor CD Hampton MB Winterbourn CC. Neutrophil NET formation with microbial stimuli requires late stage NADPH oxidase activity. Antioxidants (Basel) (2021) 10(11):1791. doi: 10.3390/antiox10111791 34829662 PMC8614658

[55] Frasch SC Fernandez-Boyanapalli RF Berry KA Murphy RC Leslie CC Nick JA. Neutrophils regulate tissue Neutrophilia in inflammation via the oxidant-modified lipid lysophosphatidylserine. J Biol Chem (2013) 288(7):4583–93. doi: 10.1074/jbc.M112.438507 PMC3576064 23293064

[56] Balci S Kisla Ekinci RM Serbes M Dogruel D Altintas DU Yilmaz M. Etanercept for the treatment of chronic arthritis related to chronic granulomatous disease: A case. Pediatr Allergy Immunol Pulmonol (2019) 32(3):131–4. doi: 10.1089/ped.2019.1036 PMC7057056 32140283

[57] Lehman HK Dave R. Candida glabrata lymphadenitis following infliximab therapy for inflammatory bowel disease in a patient with chronic granulomatous disease: case report and literature review. Front Pediatr (2021) 9:707369. doi: 10.3389/fped.2021.707369 34760850 PMC8573330

[58] Uzel G Orange JS Poliak N Marciano BE Heller T Holland SM. Complications of tumor necrosis factor-alpha blockade in chronic granulomatous disease-related colitis. Clin Infect Dis (2010) 51(12):1429–34. doi: 10.1086/657308 PMC3106244 21058909

[59] Deffert C Olleros ML Huiping Y Herrmann FR Zekry D Garcia I. TNF-alpha blockade in chronic granulomatous disease-induced hyperinflammation: patient analysis and murine model. J Allergy Clin Immunol (2011) 128(3):675–7. doi: 10.1016/j.jaci.2011.04.028 21596423

[60] Degboe Y Rauwel B Baron M Boyer JF Ruyssen-Witrand A Constantin A. Polarization of rheumatoid macrophages by TNF targeting through an IL-10/STAT3 mechanism. Front Immunol (2019) 10:3. doi: 10.3389/fimmu.2019.00003 30713533 PMC6345709

[61] Menegatti S Guillemot V Latis E Yahia-Cherbal H Mittermuller D Rouilly V. Immune response profiling of patients with spondyloarthritis reveals signalling networks mediating TNF-blocker function in vivo. Ann Rheum Dis (2021) 80(4):475–86. doi: 10.1136/annrheumdis-2020-218304 PMC7958106 33268443

[62] Mukaro VR Quach A Gahan ME Boog B Huang ZH Gao X. Small tumor necrosis factor receptor biologics inhibit the tumor necrosis factor-p38 signalling axis and inflammation. Nat Commun (2018) 9(1):1365. doi: 10.1038/s41467-018-03640-y 29636466 PMC5893557

[63] Efimov GA Kruglov AA Khlopchatnikova ZV Rozov FN Mokhonov VV Rose-John S. Cell-type-restricted anti-cytokine therapy: TNF inhibition from one pathogenic source. Proc Natl Acad Sci USA (2016) 113(11):3006–11. doi: 10.1073/pnas.1520175113 PMC4801281 26936954

