# Supplementary material for: TNFα: TNFR1 signaling inhibits maturation and maintains the pro-inflammatory programming of monocyte-derived macrophages in murine chronic granulomatous disease
Source: Front Immunol. 2024 Feb 9;15:1354836. doi: 10.3389/fimmu.2024.1354836 (PMC10884288; doi:10.3389/fimmu.2024.1354836)
Supplement: Supplementary file 1 [file DataSheet_1.docx]

**Supplementary Figure 1: Characterization of plated lavage cells**

(**A**) Composition of peritoneal lavage cells at 20h post i.p. zymosan injection was assessed by flow cytometry. Neutrophils (Neuts), resident peritoneal macrophages (RPMs), eosinophils (Eos). Mean percentage of total cells of the indicated type ± standard deviation from n>2 independent experiments with n=3 mice/genotype. Numbers in bold indicate statistically significant differences between genotypes by t-test with p<0.05. (**B**) Numbers of total live cells, MoMacs or Neutrophils recovered from wells after plating for the indicated time. Shown is the mean ± SEM representing 2 independent experiments with n=3 mice/ genotype.

**Supplementary Figure 2: Characterization of marker expression *ex vivo*.**

Graphs describe the adjusted mean fluorescence intensity (MFI) for each of the indicated phenotyping markers described in Figure 1. Data are representative of 2 independent experiments with >2 mice/ genotype. Raw MFI were adjusted to subtract unstained controls and account for differences in cytometer settings between replicate experiments. Error bars indicate mean ± SEM. t-tests were performed comparing WT and CGD at each timepoint with correction for multiple comparisons * denotes p<0.05, nd no difference.

**Supplementary Figure 3: Additional phenotyping markers tested.**

Histograms show expression of the indicated alternative MoMac phenotyping markers assessed by flow cytometry either from WT or CGD peritoneal lavage MoMacs at 20h or 72h post-zymosan or of 20h lavage MoMacs after 48h in *ex vivo* culture. Fluorescence minus one (FMO) controls were performed at each timepoint, representative controls from each genotype are shown in grey.

**Supplementary Figure 4: Characterization of marker expression *in vivo*.**

Graphs describe the adjusted mean fluorescence intensity (MFI) for each of the indicated phenotyping markers described in Figure 2. Data are representative of >2 independent experiments with 3 mice/ genotype. Raw MFI were adjusted to subtract unstained controls and account for differences in cytometer settings between replicate experiments. Error bars indicate mean ± SEM. t-tests were performed comparing WT and CGD at each timepoint with correction for multiple comparisons * denotes p<0.05, nd no difference.

**Supplementary Figure 5: Phenotyping marker expression from Figure 3.**

(**A**) Reproduced from Figure 3 for clarity: WT or CGD PL cells were treated with conditioned media (CM) from the indicated cultures. CGD conditioned media was either treated with proteinase K and boiling, or DNase I as indicated prior to addition to test cultures. tSNE was performed on pooled MoMacs after normalizing for cell numbers. (**B**) Heatmap overlays indicate the relative expression of each phenotyping marker.

**Supplementary Figure 6: Separated samples and marker expression from Figure 4**

(**A**) Relating to Figure 4C: *Top:* WT lavage cells were treated with the indicated doses of recombinant TNFα protein and tSNE analysis performed, and individual samples separated for clarity. Numbers record the % mature MoMacs from each sample. *Bottom*: Heatmap overlays indicate the relative expression of each phenotyping marker. (**B**) *Top:* Relating to Figure 4E: CGD lavage cells were treated with anti-TNFα neutralizing or isotype control antibody. tSNE analysis was performed and individual samples separated for clarity. Numbers record the % mature MoMacs from each sample. *Bottom:* Heatmap overlays indicate the relative expression of each phenotyping marker.

**Supplementary Figure 7: TNFα supplementation or neutralization does not affect neutrophil or MoMac survival *ex vivo*.**

Numbers of live (Sytox-impermeable) neutrophils or MoMacs were counted after 48 hours in culture from experiments described in Figure 4, bars show mean + SEM. WT lavage cells were treated +/- 60ng/mL recombinant mouse TNFα protein, CGD lavage cells were treated +/- TNFα-neutralizing antibody. ANOVA analysis used to identify differences: ns non-significant, **** p<0.0001.

**Supplementary Figure 8: Cytokine concentration in 48h PL culture supernatant with TNFα neutralization.**

Bar graphs show cytokine concentrations (mean ± SEM) detected in cell-free culture supernatants by multiplex array 48h after plating +/- TNFα neutralizing antibody or isotype control. Dashed line indicates the assay lower limit of detection. * p<0.05 or ** p<0.01 by ANOVA.

**Supplementary Figure 9: Neutralization of IL6, IL1β, IFNγ, or blockade of IFNAR1 has no effect on CGD MoMac maturation.**

20h post-zymosan lavage cells from CGD mice were plated in the presence of the indicated anti-cytokine or receptor blockade antibody or isotype-matched control antibody (Iso, only one example shown for clarity), further described in Table 1. tSNE analysis of MoMacs compares antibody treatment with untreated WT or CGD cells. All samples overlaid after normalizing for MoMac cell numbers (above) or shown separately for clarity (below). Numbers indicate the % mature MoMacs in each sample, defined as the cluster containing >98% of untreated WT MoMacs.

**Supplementary Figure 10: *in vivo* TNFα neutralization.**

Relating to Figure 5: CGD mice were treated with 2 doses of TNFα-neutralizing antibody at 20- and 48 hours post-zymosan before harvest at 72h. (**A**) tSNE analysis was performed on total MoMacs, individual samples shown separated for clarity. (**B**) Heatmap overlays indicate the relative expression of each phenotyping marker.

**Supplementary Figure 11: Expression of TNF receptors by MoMacs**

Expression of surface TNFR1 or TNFR2 by MoMacs from WT or CGD peritoneal lavage at 20h post-zymosan or after 48h in *ex vivo* culture was determined by flow cytometry. Data report adjusted mean fluorescence intensity (MFI, mean ± SEM) based on unstained controls of receptor staining and represent 3-5 mice per group. ANOVA was used to test for differences in expression ** denotes p<0.01, *** p<0.001, **** p<0.0001, ns = non-significant.

**Supplementary Figure 12: Separated samples and marker expression from Figure 7.**

**(A)** Relating to Figure 7A: WT or TNFR1^-/-^ cells were treated with conditioned media from CGD cells in the presence or absence of TNFα neutralizing antibody. tSNE analysis was performed and individual samples are shown separated for clarity, numbers indicate the percentage of events in the mature gate. *Bottom:* Heat map overlays indicate the expression of each phenotyping marker. **(B)** Relating to Figure 7C: WT or TNFR1^-/-^ cells were co-cultured with CGD lavage cells at a 70:30 ratio, either in favor of the CGD cells or the WT/TNFR1^-/-^ cells. tSNE analysis was performed and individual samples are shown separated for clarity, numbers indicate the percentage of events in the mature gate. *Bottom:* Heat map overlays indicate the relative expression of each phenotyping marker.

**Supplementary Figure 13: Separated samples and marker expression from adoptive transfer experiments in Figure 8.**

Relating to Figure 8: WT or TNFR1^-/-^ PL cells were adoptively transferred into CGD recipient mice at 18 hours post zymosan, mice were harvested at 72 hours post zymosan. (**A**) Total MoMacs were analyzed by tSNE analysis, individual samples are shown separated for clarity. (**B**) Heatmap overlays indicate the relative expression of each phenotyping marker.
